# An extremely rare case of Rosai–Dorfman disease in the thymus

**DOI:** 10.1186/s13019-021-01595-8

**Published:** 2021-07-31

**Authors:** Cheng Shen, Hu Liao

**Affiliations:** Department of Thoracic Surgery, West-China Hospital, Sichuan University, Chengdu, 610041 China

**Keywords:** Rosai–Dorfman disease, Thymus, Surgery, Prognosis

## Abstract

**Background:**

There were very few reports of Rosai–Dorfman disease in the thymus, which known as sinus histiocytosis with massive lymphadenopathy. It usually accompanied with other systemic symptoms such as fever, malaise, night sweats, or weight loss in the short term.

Case presentation

We herein report a rare case of RDD of thymic origin and a review of the literature concerning the clinical and pathological features of this disease, which is often misdiagnosed as thymoma. The patient was underwent thymectomy to extirpate the lesion completely by video-assisted thoracic surgery.

**Conclusions:**

To the best of our knowledge, this is the fourth thymus occurring RDD case with proof via histology and IHC. Our findings suggest the difficulty of making a diagnosis before surgery and more cases will need to be reported in order to facilitate the preoperative diagnosis of such a rare tumor.

## Introduction

Rosai–Dorfman disease (RDD) was first described by Rosai and Dorfman in 1969, accounting for 0.5% or less of all mediastinal tumors [1]. RDD disease is a non-Langerhans cell histiocytic condition more commonly seen in children and young adults, which presents fever and painless cervical lymphadenopathy [2]. We herein report a rare case of RDD of thymic origin and a review of several cases concerning the clinical and pathological features of this disease, which is often misdiagnosed as thymoma.

## Case presentation

**Fig. 1:**
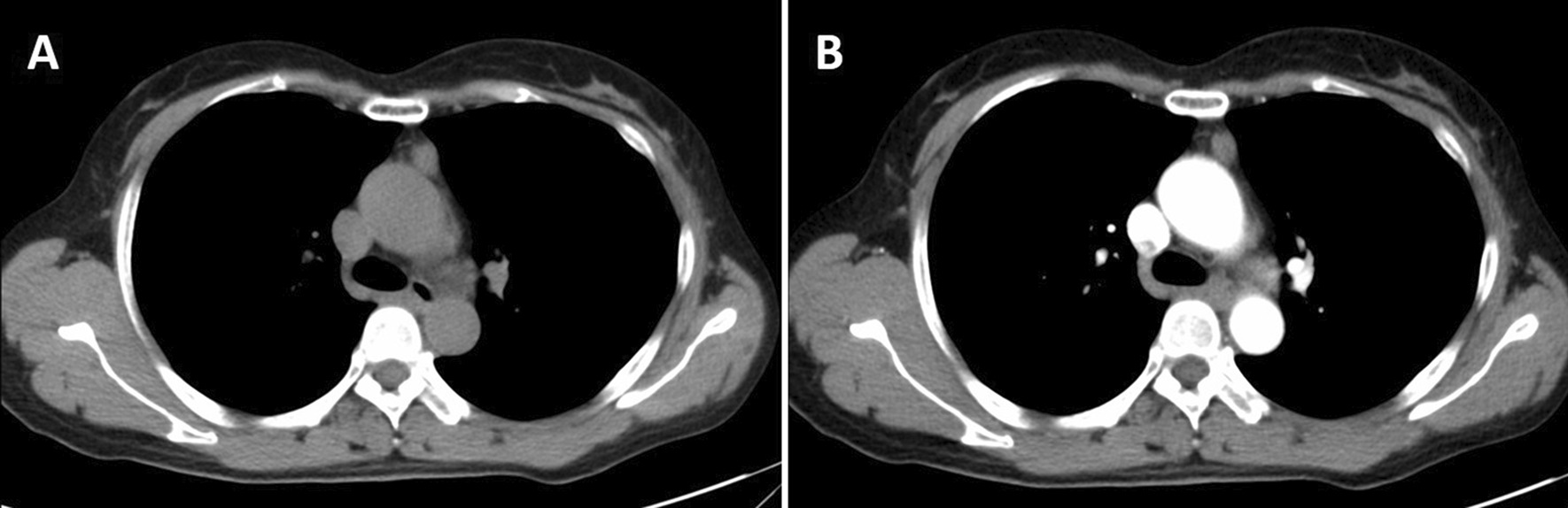
Chest contrast-enhanced CT and histological features and of the case. **a** and **b**: Contrast-enhanced CT scan showing partial enhancement in soft tissue, measuring 1.7 cm × 1.5 cm in size, in the anterior mediastinum

A 49-year-old woman was admitted to our hospital for assessment of anterior mediastinum nodule that was noticed on chest radiography during a routine health check. She had smoked one pack of cigarettes per day for the past 20 years and quit smoking for nine months. She denied the symptoms including the presence of chest pain, hoarseness, hemoptysis, cough and dyspnea. She had no risk factors for immunodeficiency disease or other infections. Physical examination shown normal breath sounds in both of chest fields. Laboratory findings were within normal limits. Her Pulmonary function tests and cardiovascular examination revealed normal performance. Plain and contrast-enhanced chest computed tomography (CT) (Fig. 1a, b) showed partial enhancement in soft tissue, calculating 1.7 cm × 1.5 cm in size, in the anterior mediastinum. As diagnosis was not established through imaging, surgery was scheduled. The patient was subjected to thymectomy by applying a three-port video-assisted thoracic surgery (VATS). There was no invasion into the adjacent structures during the surgery. After complete resection of the lesion, tissue of the mass was taken out with a biopsy forceps from the tumor for quick frozen pathology, which was pathologically diagnosed as sinus histiocytosis with massive lymphadenopathy.

**Fig. 2:**
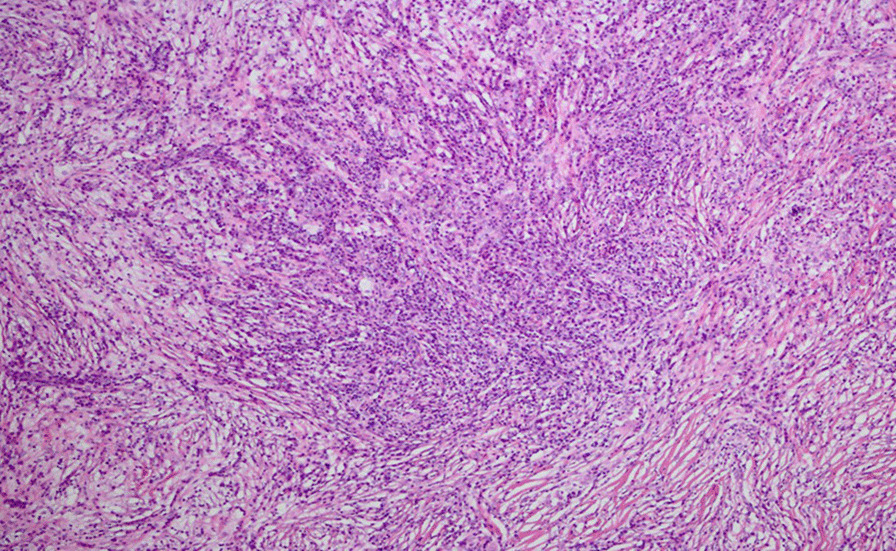
Histological features. Hematoxylin and eosin (H&E) indicated a cross distribution of deeply and lightly stained lesions. The deeply stained zone was mainly composed of a large number of plasma cells and lymphocytes, and interspersed in a flake-like, lightly stained area like stripes. The giant pleomorphic tissue cells characterized by abundant cytoplasm, vacuoles, large nuclei, and irregular nucleus were distributed in the lightly stained area. In the cytoplasm of histiocytes, emperipolesis showed as some lymphocytes and a small number of plasma cells were phagocytized

**Fig. 3:**
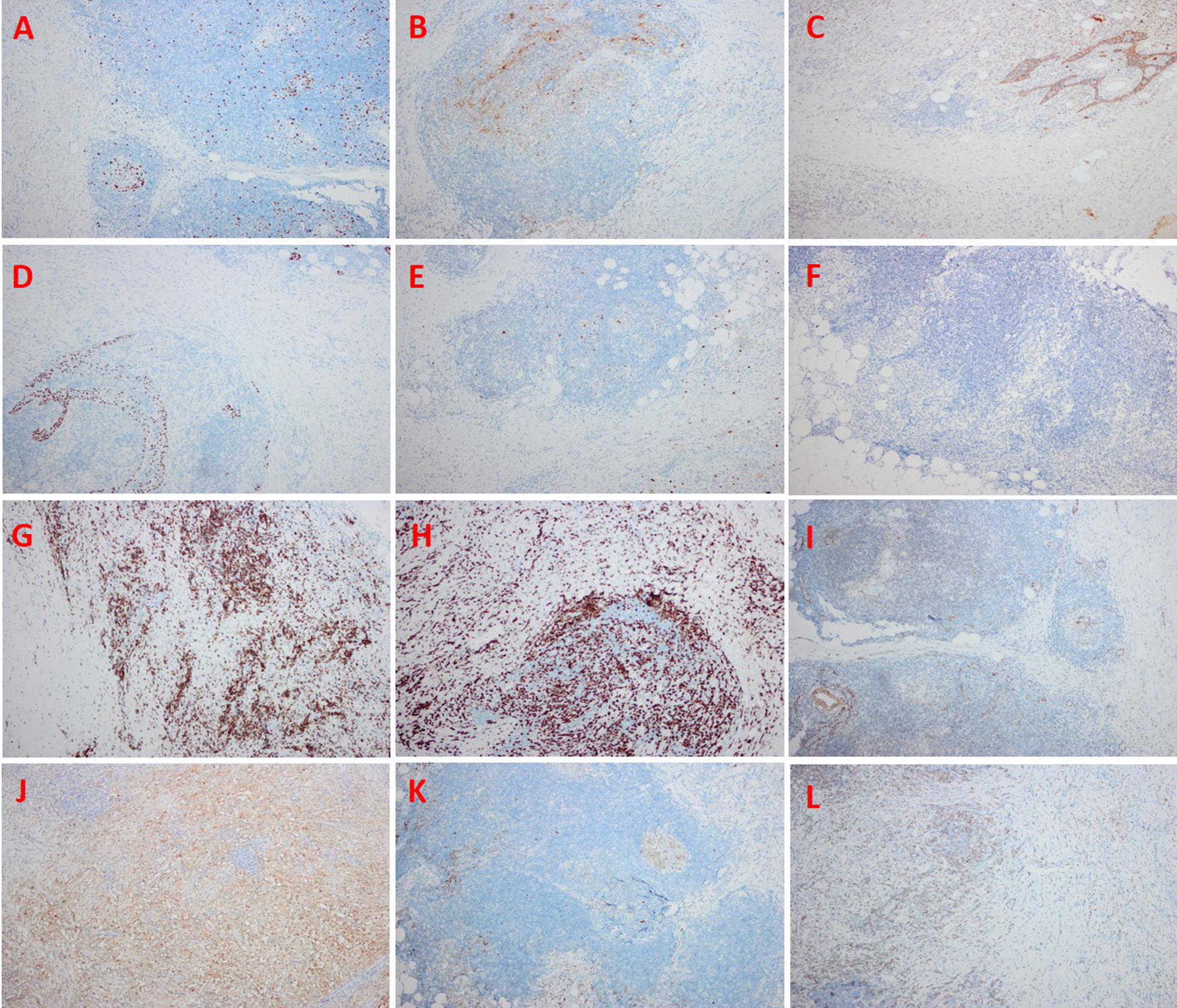
Immunohistochemistry results. S‑100 protein staining was strong positive. Thymus-associated epithelial cell indicators CK19 and P63 were positive. Cyclin D1, OCT-2, CD20, CD79a, SMA, CD30, CD10 and CD3 were also positive, and Ki67 index was about 5% in lymphocytes and plasma cells. (**a** Ki67, **b** S‑100 protein, **c** CK19, **d** P63, **e** Cyclin D1, **f** OCT-2, **g** CD20, **h** CD79a, **i** SMA, **j** CD30, **k** CD10, **l** CD3)

Hematoxylin and eosin (H&E) indicated a cross distribution of deeply and lightly stained lesions. The deeply stained zone was mainly composed of a large number of plasma cells and lymphocytes, and interspersed in a flake-like, lightly stained area like stripes. The giant pleomorphic tissue cells characterized by abundant cytoplasm, vacuoles, large nuclei, and irregular nucleus were distributed in the lightly stained area. In the cytoplasm of histiocytes, emperipolesis showed as some lymphocytes and a small number of plasma cells were phagocytized (Fig. 2). Immunohistochemically, S‑100 protein staining was strong positive. Thymus-associated epithelial cell indicators CK19 and P63 were positive. Cyclin D1, OCT-2, CD20, CD79a, SMA, CD30, CD10 and CD3 were also positive, and Ki67 index was about 5% in lymphocytes and plasma cells (Fig. 3).

The patient was discharged 2 days after the operation with no complication. She had been followed up for 3 months without evidence of recurrence.

## Discussion

RDD disease, known as sinus histiocytosis with massive lymphadenopathy (SHML), is generally a rare benign disorder consisting of a proliferation of histiocytes. There were very few reports of RDD in the thymus [3–6]. This disease can happen at any age and has a higher prevalence in males, with the mean onset age of 20.6 years [7]. Wu et al. [6] and Lim et al. [4] reported the case whose age was 42-year-old man and 43-year-old man respectively. Some studies suggest it is thought to be due to immune dysregulation or infections like HHV-6, HHV-8, parvovirus B19 and klebsiella. However, the evidence is inconclusive [8]. It also remained controversial about the association of IgG4 with RDD.

Though the clinical presentation of RDD is variable, the most common place is painless bilateral cervical lymphadenopathy [8]. It usually accompanied with other systemic symptoms such as fever, malaise, night sweats, or weight loss in the short term. Extranodal involvement was also common, and skin, upper respiratory, orbits, testes, and bones were common sites [2].

In Wu et al. report [6], the patient was admitted to facility with persistent fever that was first observed one-and-half months before the date of admission. The patient also exhibited significant fatigue and poor appetite. As showed in Lim et al. study, the patient presented with a chief complaint of a left neck mass. In our case, the patient presented without any symptoms, which is the same symptom in Cangelosi et al. [3] case description.

The radiologic diagnosis of RDD is often difficult because of the nonspecific imaging findings. Nodal involvement of RDD demonstrates massive bilateral cervical lymph node enlargement showing homogeneous enhancement on CT. MRI reveals homogeneous isointensity relative to the muscles on T1-wighted images and hyperintensity on T2-weighted images with homogenous enhancement. CT findings of extranodal RDD involving paranasal sinuses, nasal cavity, and larynx show homogeneously enhancing polypoid masses, mucosal thickening, or soft tissue opacification. In MRI images, paranasal sinus lesions may show marked T2 hypointensity. Affected sites typically show hypermetabolism of F-18 FDG on PET/CT. However, a solitary focus of increased activity in the thymus on an FDG PET/CT scan can be observed in a number of benign and malignant thymic conditions, including hyperplasia and thymoma.

The pathologic feature of nodal RDD is the sinus expansion with large histiocytes [1]. The histopathologic characteristic of RDD is the presence of lymphocytes, plasma cells, red blood cells or polymorphonuclear leukocytes within the cytoplasm of histiocytes [1]. In immunohistochemical analysis, RDD histiocytes are characterized by S100 and CD68 positivity, and when combined with lymphophagocytic histocytes [1].

The differential diagnosis of the RDD was including the xanthogranulomatous family of diseases and Langerhans cell histiocytosis (LCH). The xanthogranulomatous family of diseases can show a wide variety of morphologic characteristics, including mononuclear cells that may be vacuolated, xanthomatous, spindle-shaped and display oncocytic changes [3]. The histiocytes in LCH are characterized by irregular nuclear contours that contain Birbeck granules on electron microscopy. The associated inflammatory infiltrate is dominated by eosinophils and lymphocytes, with emperipolesis characteristically lacking [3].

The standard treatments for RDD are still unknown. Spontaneous remission is observed and “wait and watch” approach is recommended [5, 6]. Other treatments include surgical resection, chemotherapy, radiotherapy, steroids, and low dose interferon are used in several patients [2]. Very limited clinical trials have been done using chemotherapy and radiotherapy therefore the effectiveness of these methods still remains to be unclear. As showed in those cases, two patients were underwent with thymectomy by sternotomy [3, 6], the other was suffered with mediastinoscopy [4]. In our case, as the mass was in the thymus, we considered thymectomy to extirpate the lesion completely and that extirpation by VATS might be a better option to treat this condition.

To the best of our knowledge, this is the fourth thymus occurring RDD case with proof via histology and IHC. Our findings suggest the difficulty of making a diagnosis before surgery and more cases will need to be reported in order to facilitate the preoperative diagnosis of such a rare tumor.

## Data Availability

All data for this study are publicly available and are ready for the public from database of hospital.

## Abbreviations

RDD: Rosai–Dorfman disease
IHC: Immunohistochemistry
CT: Computed tomography
VATS: Video-assisted thoracic surgery
HE: Hematoxylin and eosin
SHML: Sinus histiocytosis with massive lymphadenopathy
MRI: Magnetic resonance imaging
PET/CT: Positron emission tomography/computed tomography
LCH: Langerhans cell histiocytosis
